# Droplet-based microfluidic high-throughput screening of heterologous enzymes secreted by the yeast *Yarrowia lipolytica*

**DOI:** 10.1186/s12934-017-0629-5

**Published:** 2017-01-31

**Authors:** Thomas Beneyton, Stéphane Thomas, Andrew D. Griffiths, Jean-Marc Nicaud, Antoine Drevelle, Tristan Rossignol

**Affiliations:** 1 Ecole Supérieure de Physique et de Chimie industrielles de la Ville de Paris (ESPCI Paris), CNRS UMR 8231, 10 rue Vauquelin, 75005 Paris, France; 2 CNRS, University of Bordeaux, CRPP, UPR 8641, 115 Avenue Albert Schweitzer, 33600 Pessac, France; 3 Micalis Institute, INRA, AgroParisTech, Université Paris-Saclay, 78352 Jouy-en-Josas, France; 4 Ets J. Soufflet/CRIS-OSIRIS, Quai Sarrail, BP12, 10400 Nogent-sur-Seine, France

**Keywords:** High-throughput enzymatic screening, Droplet-based microfluidics, Heterologous expression, *Yarrowia lipolytica*, Recombinant fungal protein

## Abstract

**Background:**

Droplet-based microfluidics is becoming an increasingly attractive alternative to microtiter plate techniques for enzymatic high-throughput screening (HTS), especially for exploring large diversities with lower time and cost footprint. In this case, the assayed enzyme has to be accessible to the substrate within the water-in-oil droplet by being ideally extracellular or displayed at the cell surface. However, most of the enzymes screened to date are expressed within the cytoplasm of* Escherichia coli* cells, which means that a lysis step must take place inside the droplets for enzyme activity to be assayed. Here, we take advantage of the excellent secretion abilities of the yeast* Yarrowia lipolytica* to describe a highly efficient expression system particularly suitable for the droplet-based microfluidic HTS.

**Results:**

Five hydrolytic genes from* Aspergillus niger* genome were chosen and the corresponding five *Yarrowia lipolytica* producing strains were constructed. Each enzyme (endo-*β*-1,4-xylanase B and C; 1,4-*β*-cellobiohydrolase A; endoglucanase A; aspartic protease) was successfully overexpressed and secreted in an active form in the crude supernatant. A droplet-based microfluidic HTS system was developed to (a) encapsulate single yeast cells; (b) grow yeast in droplets; (c) inject the relevant enzymatic substrate; (d) incubate droplets on chip; (e) detect enzymatic activity; and (f) sort droplets based on enzymatic activity. Combining this integrated microfluidic platform with gene expression in *Y. lipolytica* results in remarkably low variability in the enzymatic activity at the single cell level within a given monoclonal population (<5%). Xylanase, cellobiohydrolase and protease activities were successfully assayed using this system. We then used the system to screen for thermostable variants of endo-*β*-1,4-xylanase C in error-prone PCR libraries. Variants displaying higher thermostable xylanase activities compared to the wild-type were isolated (up to 4.7-fold improvement).

**Conclusions:**

* Yarrowia lipolytica* was used to express fungal genes encoding hydrolytic enzymes of interest. We developed a successful droplet-based microfluidic platform for the high-throughput screening (10^5^ strains/h) of* Y. lipolytica* based on enzyme secretion and activity. This approach provides highly efficient tools for the HTS of recombinant enzymatic activities. This should be extremely useful for discovering new biocatalysts via directed evolution or protein engineering approaches and should lead to major advances in microbial cell factory development.

**Electronic supplementary material:**

The online version of this article (doi:10.1186/s12934-017-0629-5) contains supplementary material, which is available to authorized users.

## Background

The lack of rapid and generalized high-throughput screening (HTS) systems has proven to be a major bottleneck when it comes to creating biocatalysts via directed evolution and protein engineering approaches. Currently, the most flexible and widely used HTS methods involve isolating monoclonal populations in the wells of microtiter plates. These techniques require a significant investment in terms of money, time, and space, which severely limits the number of enzymes that can be screened and, consequently, the discovery of better biocatalysts. Droplet-based microfluidics has recently emerged as an attractive alternative to conventional HTS techniques because it has significantly higher throughput and significantly lower assay volumes [1]. In this approach, cells are compartmentalized in water-in-oil (w/o) picoliter droplets that can be individually manipulated at kHz frequencies (e.g., created, fused, injected, incubated, or sorted based on fluorescence) [2–4]. Typically, single bacteria or yeast are compartmentalized in 10 pl droplets allowing the screening of libraries with a 1.000 fold-increase in speed and 1-million-fold reduction in volume compared to robotic microtiter plate-based systems [5].

Most of the time, in droplet-based microfluidic systems, enzymatic activities are detected using fluorogenic substrates especially adapted to the droplet format. In particular, the fluorescent probe product should not be transported from one compartment to another one over the time scale of the assay [6, 7], and the substrate should be accessible to the expressed enzymes within the w/o droplet. Consequently, the expression system must be carefully chosen and, ideally, should involve enzymes that are expressed extracellularly or at the cell surface. To date, most of the protein libraries screened have relied on cytoplasmic or periplasmic expression in *Escherichia coli*, which implies that both the substrate and the product travel through the cell membrane [8, 9] or that an additional lysis step is needed to perform the enzymatic assay [10–12]. While horseradish peroxidase libraries were screened displayed at the surface of *Saccharomyces cerevisiae* cells [5], none extracellular recombinant expression systems were described up to now in combination with droplet-based microfluidics.

Yeasts have the advantage of being able to perform eukaryotic post-translational processing (e.g., glycosylation, folding, sequence cleavage, etc.) and can therefore produce and secrete heterologous eukaryotic proteins in their active form. Expression and secretion of heterologous proteins have been widely studied in yeast, for which several expression systems have been commercialized. In particular, the nonconventional yeast *Yarrowia lipolytica*, has been used as a model organism for protein secretion [13–15], as it has a natural predisposition for protein secretion [16]. It is potentially better at producing heterologous proteins than are many other commonly used yeast [17], including *Pichia pastoris*, or even *E. coli* the most commonly exploited heterologous protein biofactory [18, 19]. *Y. lipolytica* presents several major advantages as a model: its genome has been fully sequenced and annotated [20]; it is a species for which numerous genetic tools [16, 21] and high-throughput cloning methods [22] are available; and an ever increasing number of its promoters and secretion signal sequences have been studied, described, and evaluated [15, 23, 24].

Moreover, there are many examples of successful heterologous protein expression using *Y. lipolytica* [25]. Because its genetic manipulation is easy to perform and its high protein yields can easily be scaled up, *Y. lipolytica* has become a cell factory of choice for producing heterologous proteins. It would thus be highly beneficial to develop a droplet-based microfluidic HTS system that uses *Y. lipolytica* because it would allow for the more efficient screening of enzyme libraries and undoubtedly advance the development of microbial cell factories in major ways.

One important aspect of enzyme production requirement today is the production of cellulolytic enzymes for the conversion of the lignocellulosic biomasses into fuels and chemicals. Cellulases are multienzyme complexes (endoglucanases, cellobiohydrolases and glucosidase) that catalyze the hydrolysis of* β*-1-4 linkages in cellulose, while xylanases cleave internal * β*-1,4 linkages of the xylan backbone. Nowadays, commercially available cellulases are produced by filamentous fungi of the genera *Trichoderma* and *Aspergillus* [26, 27]. However, improvement of catalytic efficiency, thermal stability or end-product inhibition resistance will have a great potential for sustainable biorefinery process for biomass conversion [26]. Proteases, which are used to make detergent, leather, pharmaceuticals, and food, account for more than a half of the worldwide enzyme sales and therefore represent another major industrial and commercial interest [28]. Fungal proteases are less pH sensitive and have broader substrate specificity than do bacterial equivalents. However, they exhibit lower reaction rates and heat tolerance [28]. Addressing these weaknesses should help enhance their commercial potential.

In this study, we took advantage of *Y. lipolytica*’s excellent secretion abilities to build extracellular expression systems for droplet-based microfluidic enzymatic screening applications. Five strains were constructed to produce five industrially relevant *Aspergillus niger* hydrolytic enzymes (two xylanases, one cellobiohydrolase, one endoglucanase and one aspartic protease); the enzymes were overexpressed and secreted in an active form. We developed a droplet-based microfluidic HTS system to encapsulate, cultivate, assay and sort yeast strains based on encoded enzymatic activities (10^5^ strains/h). The activity levels of the xylanases, cellobiohydrolase, and protease were successfully detected in droplets with a remarkably low variability within monoclonal populations. Combining droplet-based microfluidic technology with the *Y. lipolytica* expression system provides a highly efficient and reliable platform for the high-throughput screening of recombinant enzyme activities.

## Results and discussion

### Expression of fungal genes in *Yarrowia lipolytica*

**Fig. 1:**
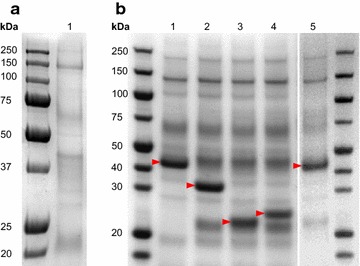
Fungal gene expression SDS-PAGE profiles of producing strains. **a** Genetic background.* Lane 1*, crude supernatant from empty strain JMY2810; **b** producing strains.* Lanes* *1*–*5*, crude supernatant from respectively strains JMY4407 (aspartic protease; 41 kDa), JMY4510 (endo-*β*-1,4-xylanase C; 33.5 kDa), JMY4343 (endo-*β*-1,4-xylanase B; 22 kDa), JMY4346 (endoglucanase A; 26 kDa) and JMY4469 (1,4-*β*-cellobiohydrolase A; 48 kDa)

Five genes from *Aspergillus niger* (CBS513.88 strain [29]) encoding industrially relevant hydrolytic enzymes were used: *xlnb* (endo-*β*-1,4-xylanase B), *xlnc* (endo-*β*-1,4-xylanase C), *cbha* (1,4-*β*-cellobiohydrolase A), *egla* (endoglucanase A), and *pep1* (aspartic protease). The five genes were codon optimized and overexpressed in *Y. lipolytica* strain JMY2566 (see "Methods" section). This strain possesses a zeta docking platform that favors integration at that specific locus [22, 30], which allows for the efficient comparison of enzymatic activity among strains. The five resulting producing strains—JMY4346 (*xlnb*), JMY4510 (*xlnc*), JMY4469 (*ccbha*), JMY4343 (*egla*) and JMY4407 (*pep1*)—were cultivated in 20 ml of YNB glucose medium for 24 h and the corresponding supernatants were profiled using SDS-PAGE (Fig. 1). Each of the target enzymes was successfully overproduced and secreted as a major protein in the crude supernatant. All the enzymes demonstrated high expression levels without requiring any extensive optimization of growth conditions.

### Enzymatic activity of the secreted proteins

**Fig. 2:**
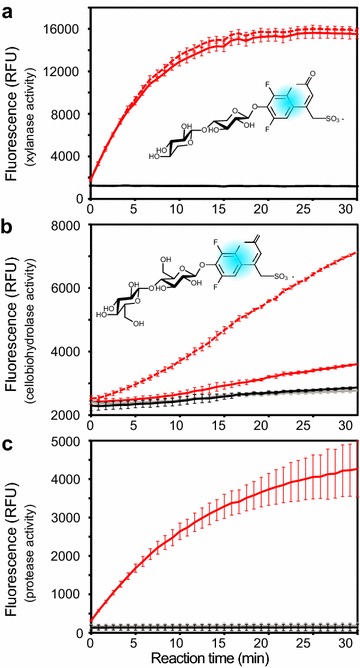
Hydrolytic activities of crude supernatants. **a** Xylanase assay performed in 50 mM acetate buffer pH 4.5 containing 150 μM of xylobiose-based substrate for strains JMY4510 (*xlnc*,* red dotted line*) , JMY4346 (*xlnb*,* red continuous line*), JMY4407 (*pep1*,* black line*) and a YNB blank (*gray line*). **b** Cellulase assay performed in 50 mM acetate buffer pH 4.5 containing 150 μM of cellobiose-based substrate for strains JMY4469 (*cbha*,* red dotted line*), JMY4343 (*egla*,* red continuous line*), JMY4407 (*pep1*,* black line*) and a YNB blank (*gray line*). **c** Protease assay performed in 50 mM acetate buffer pH 4.0 containing 25 μg/l of Enzchek Protease substrate for strains JMY4407 (*pep1*,* red line*), JMY4510 (*xlnc*,* black line*) and a YNB blank (*gray line*)

To determine if the encoded proteins were produced in an active form, the xylanase, cellulase or protease activity was assayed in crude supernatants using fluorogenic substrates (Fig. 2). The xylanase and cellulase substrates were synthesized as previously described in [31], using a sulfonated 7-hydroxycoumarin scaffold linked to a saccharide recognition unit. Xylanase activity was detected by monitoring the hydrolysis of a xylobiose-based coumarin (*β*-d-xylobioside-6,8-difluoro-7-hydroxycoumarin-4-methansulfonate, inset Fig. 2a) while cellulase (i.e., cellobiohydrolase) activity was detected by monitoring the hydrolysis of a cellobiose-based coumarin (*β*-d-cellobioside-6,8-difluoro-7-hydroxycoumarin-4-methansulfonate, inset Fig. 2b). The protease substrate is commercially available and consists of a casein scaffold labeled with quenched green-fluorescent BODIPY^®^FL fluorophores. Protease-catalyzed hydrolysis releases the fluorophores, revealing enzyme activity. All encoded proteins were found to be active after secretion.

The JMY4346 (*xlnb*) and JMY4510 (*xlnc*) strains exhibited similar levels of endo-*β*-1,4-xylanase activity, corresponding to endo-*β*-1,4-xylanase B and endo-*β*-1,4-xylanase C, respectively (Fig. 2a). Strain JMY4469 (*cbha*) displayed cellobiohydrolase activity corresponding to 1,4-*β*-cellobiohydrolase A (Fig. 2b). While the cellulase substrate was designed to reveal cellobiohydrolase activity, JMY4343 (*egla*) displayed a low activity with this substrate, seemingly due to the endocellulase activity of endoglucanase A (Fig. 2b). Lastly, strain JMY4407 (*pep1*) showed protease activity corresponding to aspartic protease (Fig. 2c). These results indicate that each of the five fungal hydrolytic genes was successfully overexpressed and that the corresponding recombinant enzymes were secreted in an active form.

### Droplet-based microfluidic HTS platform

**Fig. 3:**
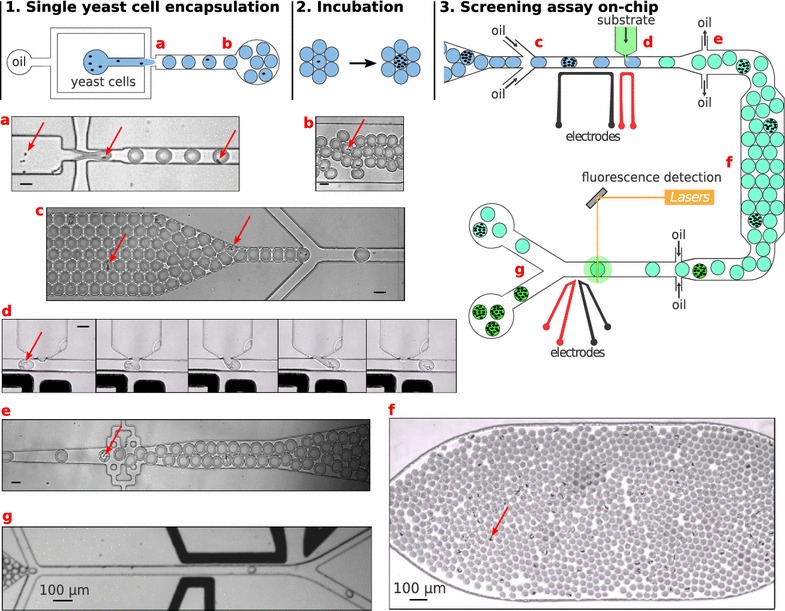
Droplet-based microfluidic screening platform. 1. Single cell encapsulation: yeast cells were encapsulated in 20 pl droplets at 1200 droplets/s by flow-focusing of a cell suspension with two streams of Novec7500 oil containing 2.5% (w/w) KryJeffD surfactant. Droplets were collected off-chip in a glass vial. 2. Cell growth and enzyme secretion: droplets were incubated for 16 h at 28 °C. 3. Enzymatic screening: droplets were loaded into an integrated device and spaced using Novec7500 oil containing 2.5% (w/w) KryJeffD_900_ surfactant. To initiate the enzymatic reaction, a fluorogenic substrate was picoinjected in each droplet at a rate of 300 droplets/s by applying an AC field (20 kHz, 200 V_*pp*_). Droplets were incubated on-chip along a delay line and spaced with Novec7500 oil for fluorescence detection (i.e. enzymatic activity). Droplets could be sorted based on fluorescence at 300 droplets/s by applying AC field pulses (30 kHz; 1200 V_*pp*_; 0.3–0.6 ms).** a**–**f** Microscopic images of the different steps of the microfluidic system. The* red arrows* indicate encapsulated *Y. lipolytica* cells. Unless specified,* scale bars* are 30 μm

A droplet-based microfluidic system was developed for the enzymatic screening of recombinant proteins secreted by *Y. lipolytica* (Fig. 3). The platform was composed of two distinct microfluidic devices: a dropmaker and an integrated screening device. The dropmaker encapsulated single yeast cells in 20-pl droplets (Additional files 1, 2 and 3). The cells were grown in the droplets off-chip for 16 h at 28 °C. The droplets were then loaded in an integrated screening device (Additional files 4 and 5) to: (1) picoinject [32] the fluorogenic substrate (Additional file 6), (2) incubate the droplets on-chip as the enzymatic reaction took place (Additional file 7) and (3) analyze the fluorescence (i.e., enzymatic activity) of each droplets, which could then be sorted based on fluorescence [33].

#### Encapsulation and growth of *Y. lipolytica* in 20 pl droplets

Droplet-based microfluidic approaches have previously been used with *S. cerevisiae* [5, 34–37] but never with *Y. lipolytica*. The encapsulation procedure had to be optimized to achieve reliable single cell encapsulation. First of all, microfluidic channels are usually treated to be both hydrophobic and fluorophilic to have proper surface channel wettability for droplets formation. However, *Y. lipolytica* displays an affinity for hydrophobic substrates [38] and tended to adhere to and aggregate on channel walls when flowed in the dropmaker device. To prevent adhesion, cell suspensions were supplemented with 0.1% TWEEN^®^20 non-ionic detergent. In addition, tubing with a small inner diameter (100 μm)was used to increase shear stress and prevent the formation of cell aggregates prior to encapsulation. The cell suspensions were also continuously stirred during the encapsulation process to prevent cell sedimentation and variation in local cell density. Following these modifications, single *Y. lipolytica* cells were successfully encapsulated in 20 pl droplets. Droplets were produced at a rate of 2500 to 3000 per second via hydrodynamic flow focusing [39] of a cell suspension with a fluorinated oil phase containing 2.5% w/w KryJeffD_900_ fluorosurfactant [40]. The number of yeast cells per droplet followed a Poisson distribution [41] and was controlled by adjusting the initial density of the cell suspensions to give an average number of cells per droplets,* λ*, of 0.03–0.1 (Additional file 8). Droplets were collected off chip in a glass vial and incubated for 16 h at 28 °C to allow cell growth and enzyme secretion. Based on image analysis, the droplets contained dozens of cells after incubation, showing that *Y. lipolytica* was able to replicate in the droplets.

#### Detection of enzymatic activities in droplets

**Fig. 4:**
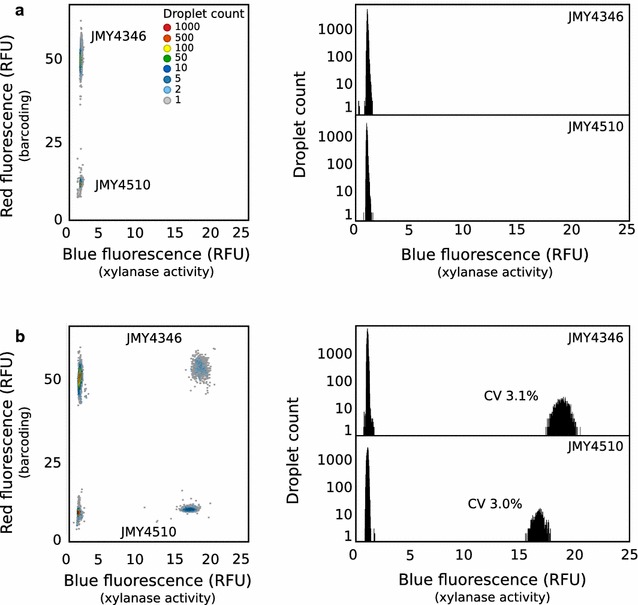
JMY4346 ( *xlnb*) and JMY4510 ( *xlnc*) strains activity in droplets 2D histograms showing the blue fluorescence (xylanase activity) and the red fluorescence (barcoding) of a binary 20 pl droplets emulsion containing JMY4346 (*xlnb*) and JMY4510 (*xlnc*) strains analyzed in the integrated screening device just after injection of the xylanase substrate (**a**) and after on-chip incubation (**b**). On the* right* are displayed the 1D histograms showing the number of droplets observed as a function of the xylanase activity (blue fluorescence) corresponding to each droplet population (JMY4510 (*xlnc*) and JMY4346 (*xlnb*) strains)

In the first set of experiments, the two xylanase strains JMY4346 (*xlnb*) and JMY4510 (*xlnc*) were assayed. Two strain-specific emulsions were produced sequentially with a* λ* equal to 0.03. The strains were encapsulated in growth medium containing strain-specific concentrations of sulforhodamine B red fluorophore to optically encode droplets (10 and 50 μM for JMY4510 [*xlnc*] and JMY4346 [*xlnb*], respectively). The binary emulsion was collected in a glass vial, strains were grown for 16 h at 28 °C, and the droplets were then loaded into the integrated screening device. The xylobiose-based coumarin substrate was picoinjected into every droplet, and fluorescence was measured immediately after injection (t_0_, Fig.4 a) and after 10 min of on-chip incubation (t_*outlet*_, Fig.4 b). As expected, both strains displayed xylanase activity. For JMY4346 (*xlnb*) strain, a positive droplet population showing xylanase activity (2.7% of the droplets [theoretically 3% if* λ* = 0.03]; RFU: 18.4 ± 3.1%) clearly demarcated from the empty droplet main population (RFU: 1 ± 5.9%). In the same way, for JMY4510 (*xlnc*) strain, a positive population (2.4% of the droplets; RFU: 16.4 ± 3%) was well separated from the empty droplet population (RFU: 1 ± 7.1%). Both strains showed remarkably low variability in their signal distributions (coefficient of variation [CV] of 3%). This narrow phenotypic distribution at the single cell level within a monoclonal population is highly desirable for enzymatic screening applications and is due to a combination of two factors. First, the expression system developed in *Y. lipolytica* under a strong constitutive promoter is characterized by low variability in expression levels [42]. Second, the integrated screening device allows for a high degree of control. The injection of the substrate to initiate the enzymatic reaction and the on-chip incubation along the delay line insure a precise control of the incubation time in each droplet. More specifically, the delay line structure design, made of constrictions and expansions, ensured that average droplet residence time, and hence incubation time, displayed little variance [43].

**Fig. 5:**
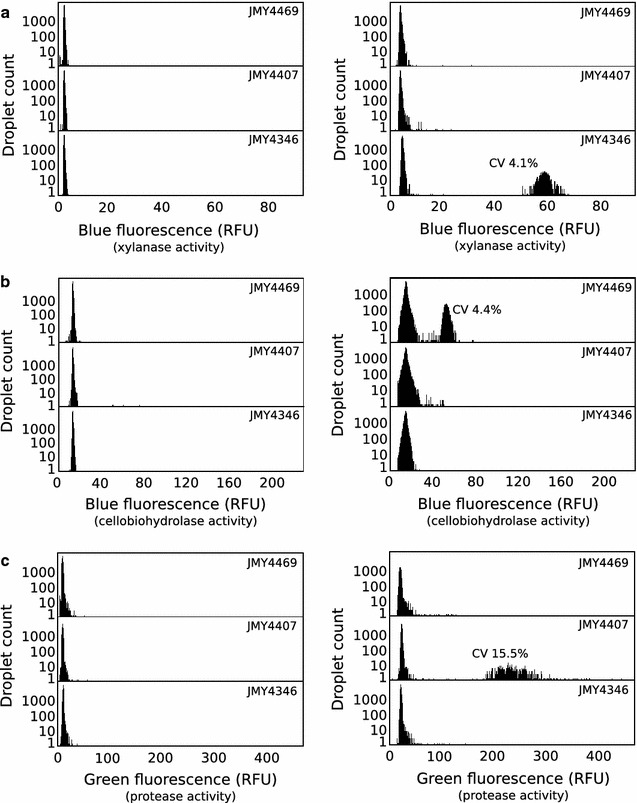
JMY4346 (*xlnb*), JMY4469 (*cbha*) and JMY4407 ( *pep1*) strains activity in droplets 1D histograms showing the number of droplets observed as a function of the enzymatic activity (*blue* or* green* fluorescence) for a ternary emulsion containing JMY4346 (*xlnb*), JMY4469 (*cbha*) and JMY4407 (*pep1*) strains. The emulsion was analyzed in the integrated screening device just after injection of the relevant enzymatic substrate (*left*) and after on-chip incubation (*right*) with the xylanase substrate (**a**), the cellulase substrate (**b**) and the protease substrate (**c**)

In a second set of experiments, strains JMY4346 (*xlnb*), JMY4469 (*cbha*), and JMY4407 (*pep1*) were assayed for xylanase, cellobiohydrolase, and protease activity, respectively. Three strain-specific emulsions were produced sequentially with a* λ* equal to 0.04, 0.08 and 0.03 for JMY4346 (*xlnb*), JMY4469 (*cbha*), and JMY4407 (*pep1*), respectively. The strains were encapsulated in growth medium containing strain-specific concentrations of sulforhodamine B red fluorophore to optically encode droplets (10, 50 and 100 μM for JMY4346 [*xlnb*], JMY4407 [*pep1*], and JMY4469 [*cbha*], respectively). Following off-chip incubation, the droplets were loaded into the integrated screening device and were picoinjected with either the xylobiose-based coumarin substrate (Fig.5a), the cellobiose-based coumarin substrate (Fig.5b), or the protease substrate (Fig.5c). Each strain showed hydrolytic activity against the respective substrate with comparable narrow signal distribution, while no activity was detected for the other strains.

These results demonstrate that our droplet-based microfluidic HTS system is an efficient and reliable method for detecting endoxylanase, cellobiohydrolase, and protease activity. The low variability obtained within monoclonal populations makes this approach very useful for screening enzyme libraries in the context of protein engineering or directed evolution.

### Screening for thermostable mutants of the endo-*β*-1,4-xylanase C

**Fig. 6:**
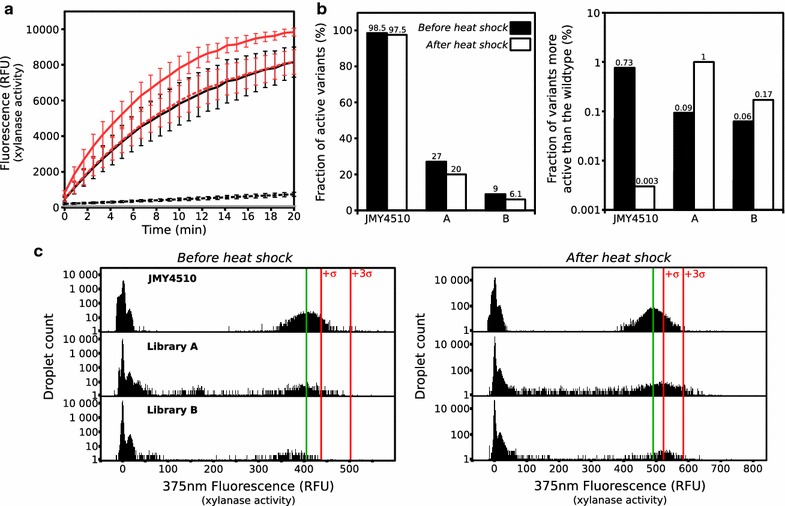
Screening for thermostable variants of endo-*β*-1,4-xylanase C. **a** Thermostability of endo-*β*-1,4-xylanase B and C. Xylanase activity of the crude supernatant for the strains JMY4510 (*xlnc*;* black line*) and JMY4346 (*xlnb*;* red line*) before (*continuous line*) and after (*dotted line*) a heat shock (90 °C, 30 s). The negative control (i.e., growth medium) is shown in* gray*.* Error bars* correspond to ±1 standard deviation (*N = 3*). **b** Microfluidics library analysis. The two libraries A and B and the parental strain (JMY4510 (*xlnc*)) were analyzed using the integrated microfluidic screening device. The histograms show the general percentage of variants displaying activity (*left*) and the proportion of variants with activity levels greater than of the parental strain (*right*) for the wild-type and each library before (*black*) and after (*white*) heat shock (90 °C, 30 s). **c** 1D histograms showing the number of droplets observed as a function of the xylanase activity before and after heat shock for the wild-type, library A and library B. The mean signal for the wild-type is indicated with a* green line*, and values* σ* and >3* σ* are to the right of the* red lines*. Strains were considered to be positives when displaying a xylanase activity higher than (mean wild-type activity + 3* σ*)

Improving the thermostability of the enzymatic cocktail composed of endo-*β*-1,4-xylanase B and C would be of particular interest from a biotechnological perspective in the field of animal feed. The thermostability of both xylanases against a heat shock of 90 °C for 30 s was investigated (Fig.6a). Crude supernatant from strains JMY4346 (*xlnb*) and JMY4510 (*xlnc*) were assayed in bulk with the xylanase substrate before and after the heat shock. Endo-*β*-1,4-xylanase C was found to be less thermostable than endo-*β*-1,4-xylanase B with residual activities of 3.7 and 71.7%, respectively.

We therefore focused on improving endo-*β*-1,4-xylanase C thermostability and set up a screen for thermostable variants as proof of concept of our droplet-based microfluidic HTS system. Libraries were generated from *xlnc* gene using error-prone PCR. To evaluate how mutation frequency affects a library phenotypic distribution, we constructed two small libraries with different mutation frequencies: library A had a low mutation frequency (2 per kb) and library B had a medium mutation frequency (>4 per kb). Gene libraries were transformed in *Y. lipolytica*, yielding 800 clones for library A and 600 clones for library B. Using the droplet-based microfluidic HTS system described above, both libraries were analyzed before and after a heat shock of 90 °C for 30 s (Fig.6b, c; Additional file 9). Before exposure to heat shock, 27% of the clones (216 clones) in library A and 9% of the clones (54 clones) in library B displayed xylanase activity. None of these clones were found to have greater activity than wild-type xylanase (>[mean wild-type activity + 3*σ*]). Those library phenotypic distributions were associated with mutation frequency: the higher mutation rate led to a lower number of active clones. After exposure to heat shock, 1% of the clones (8 clones) in library A were found to have higher activities than the wild-type xylanase (>[mean wild-type activity + 3*σ*]), while this percentage was 0.17% (1 clone) for library B. Therefore, our droplet-based microfluidic HTS approach revealed that 8 and 1 potential thermostable variants could be found in library A and B respectively.

To evaluate the sensitivity of our droplet-based microfluidic HTS system as compared to more classical, lower-throughput microtiter plate methods, 480 clones were isolated from library A and placed in 96-well plates. Each was individually screened for xylanase thermostable activity. As above, prior to the heat shock, none of the clones were found to have higher levels of activity than wild-type xylanase (>[mean wild-type activity + 3*σ*]; data not shown). Following the heat shock, six clones demonstrated activity greater than that in the wild-type (>[mean wild-type activity + 3*σ*]), which corresponded to 1.25% of the library. These results are very similar to those obtained using the droplet-based microfluidic HTS approach. The six mutants with thermostable xylanase were individually grown in test tubes and enzyme activity levels in the supernatants were evaluated in duplicate before and after a heat shock (Additional file 10). All the mutants had an absolute xylanase activity level higher than that of the wild-type after a heat shock. Four of the six clones had more than 30% residual activity; the wild-type had only 5%. The sequencing of the mutated gene in the six clones reveals that they contain from 1 to 6 point mutations (average of 2.5) at the amino acid level (Additional file 11). Mutations are located all along the protein, with a higher density at the N-terminus (Additional file 11). Thus, the thermoresistance observed for these clones is likely to be a consequence of these mutations.

## Conclusion

These results show that using a droplet-based microfluidic HTS approach with the *Y. lipolytica* expression system allowed for a rapid, efficient, and reliable kinetic analysis of recombinant enzymes at the single cell level. This study focused on the expression of fungal genes encoding hydrolytic enzymes of biotechnological interest, but the technique could be extended to any gene encoding for an enzymatic activity that could be detected using a fluorescent assay compatible with droplet-based microfluidics. Another application will be the screening of strains having altered metabolisms using fluorescent reporter systems. We have developed a precise, high-throughput and low-cost screening technique that can be used to compare and isolate improved enzymes within libraries. This will undoubtedly help to speed up the discovery of new enzymes via directed evolution or protein engineering strategies.

## Methods

### Strains and plasmids

The five fungal heterologous genes from *A. niger* strain CBS 513.88—*xlnb* (endo-*β*-1,4-xylanase B), *xlnc* (endo-*β*-1,4-xylanase C), *cbha* (1,4-*β*-cellobiohydrolase A), *egla* (endoglucanase A), and *pep1* (aspartic protease)—were synthesized with codon optimization by GenScript (Piscataway, NJ, USA) and in accordance with the codon usage in *Y. lipolytica*. Codon optimized genes were cloned into a JMP62-type vector [13, 44] under the control of the strong and constitutive pTEF promoter [17]. The vectors used in this study are listed in Additional file 12. Expression vectors were digested with the restriction enzyme NotI (New England Biolabs, Evry, France) to release the expression cassettes. The gel-purified expression cassettes were then used to transform *Y. lipolytica* strain JMY2566 [22] using the lithium acetate method [45], giving rise to strains JMY4346 (*xlnb*), JMY4510 (*xlnc*), JMY4469 (*cbha*), JMY4343 (*egla*), and JMY4407 (*pep1*). Transformants were selected on YNB plates. Genomic DNA from the positive transformants was prepared as described elsewhere [46], and integration of the overexpression cassette at the specific zeta locus was confirmed by PCR using gene-specific primers (Additional file 13) and a primer located in the zeta locus (5’-TCTTCTGCCTCCAGGAAGTC-3’). DNA fragments were recovered from agarose gels using a QIAquick Gel Extraction Kit (Qiagen, Hikden, Germany). The QIAprep Spin Miniprep Kit (Qiagen, Hilden, Germany) was used for plasmid purification. All the reactions were performed in accordance with the manufacturer’s instructions.

### Growth conditions

*Escherichia coli* strains were grown according to standard protocols [47] in Luria-Bertani broth medium containing 50 μg/ml kanamycin. For the yeast inoculum, rich YPD medium containing 1% (w/v) yeast extract, 1% (w/v) peptone, and 2% (w/v) glucose was used. Transformants were selected on minimal YNB medium, composed of 0.17% (w/v) yeast nitrogen base (without amino acids and ammonium sulfate; BD Difco), 0.5% (w/v) NH4Cl, 50 mM phosphate buffer (pH 6.8), and 2% (w/v) glucose. When necessary, leucine and uracil were added to a final concentration of 0.1 g l. Solid media were complemented with 1.6% agar.

Single colonies were grown for 24 h at 28 °C in 2 ml of YNB glucose medium (YNB without amino acids [1.7 g/l], yeast extract [1.5 g/l], glucose [10 g/l], NH4Cl [100 mM], and KH_2_P0_4_/Na_2_HPO_4_ buffer [50 mM, pH 6.8]). 20 ml of fresh YNB glucose medium were inoculated at an OD_600_ value of 0.05. The culture was grown at 28 °C until it reached an OD_600_ value of 0.4–0.6, in the microfluidic experiments, or for 24 h for bulk measurements of hydrolytic activities and SDS analysis.

In the mutant library experiment, fresh YNB glucose medium was inoculated with −80 °C glycerol stock cell suspensions with an OD_600_ value of 0.05.

For growth in 96-well plates, yeasts were precultivated for 48 h in YPD medium in microtiter plates. 5 μl of the precultures were then transferred to 120 μl of fresh YNB medium and grown for 30 h at 28 °C on an orbital shaker at 170 rpm.

### SDS-PAGE analysis

20 μl of crude supernatant were analyzed by electrophoresis on a 10% NuPAGE Bis-Tris-acetate sodium dodecyl sulfate-polyacrylamide gel (SDS-PAGE) (Thermo Fisher Scientific, Villebon-sur-Yvette, France). After migration, the gel was subject to colloidal blue staining.

### Primary enzymatic activity detection on yeast transformants

Cells were grown for 24 h and then removed via centrifugation (6000 rpm for 20 min, 28 °C). Xylanase, cellulase, or protease activity levels in the crude supernatants were monitored using adapted fluorescence assays in a Spectramax M5 spectrofluorometer (Molecular Devices, Wokingham, UK).

#### Xylanase assay

25 μl of crude supernatant was added to 25 μl of a 300 μM solution of* β*-d-xylobioside-6,8-difluoro-7-hydroxycoumarin-4-methansulfonate in acetate buffer (100 mM, pH 4.5). The reaction was monitored at 25 °C for 30 min (*λ*_ex_ = 360 nm,* λ*_em_ = 460 nm).

#### Cellobiohydrolase assay

25 μl of crude supernatant was added to 25 μl of a 300 μM solution of* β*-d-cellobioside-6,8-difluoro-7-hydroxycoumarin-4-methansulfonate in acetate bufffer (100 mM, pH 4.5). The reaction was monitored at 25 °C for 30 min (*λ*_ex_ = 360 nm, *λ*_em_ = 460 nm).

#### Protease assay

25 μl of crude supernatant was added to 25 μl of a 50 μg/ml solution of Enzchek Protease Assay substrate (Thermo Fisher Scientific, Villebon-sur-Yvette, France) in acetate buffer (100 mM, pH 4.0). The reaction was monitored at 25 °C for 30 min (*λ*_ex_ = 480 nm, *λ*_em_ = 518 nm).

### Droplet-based microfluidics

#### Fabrication of microfluidic devices

Poly-(dimethylsiloxane) (PDMS) microfluidic devices were fabricated as previously described [8] from 20 or 70 μm-deep molds of SU-8 2025 and SU-8 2050 negative photoresists (MicroChem Corp), respectively.

#### Optical setup, data acquisition and control system for droplet analysis

The optical setup used to monitor microfluidic experiments has previously been described, as well as the data acquisition and control system [31]. In addition, to allow the sorting of a particular droplet, the data acquisition card provided a signal to a model 623B high-voltage amplifier (Trek Inc.) connected to the electrodes of the microfluidic device.

#### Surfactant synthesis

We used aqueous droplets in Novec7500 fluorinated oil (3M) stabilized against coalescence by a triblock copolymer fluorosurfactant comprising two perfluoropolyether (PFPE) chains linked by one Jeffamine^®^ polyetheramine chain (PEA), KryJeffD_900_. KryJeffD_900_ surfactant was prepared in house as previously described [40].

#### Microfluidic chip operation

Droplets were produced using a dropmaker device (Additional file 1) by flow-focusing the cell suspension stream with two streams of Novec7500 oil containing 2.5% (w/w) of KryJeffD_900_ surfactant. The liquids were pumped into the dropmaker using standard infusion/withdraw PHD 22/2000 syringe pumps (Harvard Apparatus Inc.). For the oil steams, a syringe (Injekt^®^; BBRAUN) was connected to the dropmaker using a 0.6 × 25 mm needle (Terumo) and PTFE tubing (Fisher Scientific) with an inner diameter (ID) of 0.56 mm and an outer diameter (OD) of 1.07 mm. For the cell suspension stream, a glass syringe (Hamilton Gastight 1002) was connected to the dropmaker using 1/32” fluidic connections with FEP tubing (ID 102 μm, OD 0.79 mm). The dropmaker was used to produce 20 pl droplets (Q_*aqueous*_ 150 μl/h, Q_*oil*_ 400 μl/h, 2000–3000 droplets/s). The droplets flowed off chip through PTFE tubing (IDEX) to a glass vial attached to a Peltier device, where they were incubated at 28 °C [48]. The droplets were then loaded into the integrated screening device (Additional file 4). The liquids were pressure driven into the device via PTFE tubing (ID 0.56 mm, OD 1.07 mm) using a microfluidic flow control system (MFCS [2 × 2 bar inlets, 2 × 1 bar inlets], Fluigent). Droplets were reloaded (P_*droplets*_ 500–700 mbar) and spaced with Novec7500 oil (P_*oil*_ 500–700 mbar). The substrate was picoinjected (P_*substrate*_ 500–700 mbar, 200–300 droplets/s) by applying an AC field (20kHz, 200 V_*pp*_), and the droplets were incubated on chip after oil extraction (Q_*extrac*_ −25 to −100 μl/h). The droplets were spaced after incubation (Q_*spacing*_ 200–1000 μl/h) and analyzed by the optical setup. Fluorescent droplets could be sorted at a rate of 300 droplets/s by applying a AC field pulses (30 kHz; 1200 V_*pp*_; 0.3–0.6 ms).

### Strain activity in droplets

#### Yeast suspension

20 ml of fresh YNB glucose medium was inoculated at an OD_600_ value of 0.01. The strains were cultured at 28 °C until an OD_600_ value of 0.4–0.6 was reached. 15 ml of culture were then centrifuged (6000 rpm, 10 min, 4 °C), and the resulting pellet was washed with fresh YNB glucose medium. This washing step was repeated three times, and the pellet was resuspended in 1 ml of fresh YNB glucose containing 0.1% TWEEN^®^20 and 10 μM sulforhodamine B (barcoding dye). The OD_600_ was measured, and the yeast suspension was then diluted to achieve the desired number of cells per droplet volume (an OD_600_ of 1 corresponds to 3.4 × 10^7^ cells per ml).

#### Yeast encapsulation

The cell suspension was encapsulated in 20-pl droplets using the dropmaker. A cylindrical PTFE magnetic stirring bar was used to avoid cell sedimentation in the syringe during the encapsulation process. The droplets were collected in a glass vial. For multiple strains emulsions, the strains were encapsulated sequentially using different level of coding dye and collected together in a single glass vial. The yeasts were cultured in the droplets at 28 °C for 24 h.

#### Assays in droplets

The droplets were loaded into the integrated screening device, and the substrate was picoinjected into every droplets (typically 5 pl in 20 pl droplets). To assay xylanase activity, the xylobiose-based substrate (150 μM in acetate buffer [50 mM, pH 4.5]) was used. To assay cellobiohydrolase activity, the cellobiose-based substrate (150 μM in acetate buffer [50 mM, pH 4.5]) was used. Fluorescence was then exited using a 375 nm laser. To assay protease activity, the Enzchek Protease Assay substrate (150 μg/ml in acetate buffer [50 mM, pH 4.0]) was used. Fluorescence was then exited using a 488 nm laser. Droplet fluorescence was measured just after injection (reaction initiation, t_0_) and after on-chip incubation (10 min at room temperature, t_*outlet*_).

### Screening for thermoresistant xylanase

#### Construction of error prone *xlnc* mutant libraries

The expression cassette from plasmid JME2603 (Additional file 12) is flanked by zeta regions and is composed of a URA3 marker, the pTEF promoter, and the *xlnc* gene (Additional file 14). We built libraries of error-prone *xlnc* variants using a two-step PCR amplification according to [30]. First, the PCR fragment composed of Zeta1, the URA3 marker, and the pTEF promoter was amplified using Zeta1for (5’-GCGGCCGCTGTCGGGAACCG-3’) and ep-PCRrev (5’-CCGATCCTTCGGGTGTGAGTTGACAAGGAG-3’) as primers and JME2603 as a template under standard conditions (100 pmoles of each primer, 5 ng of DNA template, 0.25 mM dNTP, 1U Pyrobest [Takara], and 1X Pyrobest buffer in a final 50 μl), which produced a 2037-bp fragment (Additional file 14 PCR1). Then, the PCR fragment composed of *xlnc* and Zeta2 was amplified using epPCRfor (5’-CTCCTTGTCAACTCACACCCGAAGGATCGG-3’) and Zeta2rev (5’-GCGGCCGCACTGAGGGCTTTG-3’) as primers and JME2603 as a template under mutagenic conditions (epPCR), which produced a 1557-bp fragment (Additional file 14; PCR2). A low-fidelity Taq DNA polymerase (NEB) was used, as per standard procedure; we also added either 1 or 2 μl of a dNTP mutation mix (8 mM dTTP, 8 mM dCTP, 96 mM MgCl_2_, and 10 mM MnCl_2_ in water) to generate a low mutation rate library A or a medium mutation rate for library B, respectively. Primers epPCRrev and epPCRfor are reverse and complementary. The PCR fragments were recovered from agarose gels using a QIAquick Gel Extraction Kit (Qiagen). A fusion PCR was then performed under standard conditions using Zeta1for and Zeta2rev as primers and an equimolar proportion of both fragments as templates (Additional file 14; PCR3). The resulting PCR fragments containing the full mutated expression cassette were used to transform *Y. lipolytica* JMY2566 as described in the section on strain and plasmid.

#### Library analysis in microfluidics

The JMY4510 (*xlnc*) wild-type, library A, and library B were encapsulated in 20 pl droplets as described above. After 16 h of incubation at 28 °C, the droplets were loaded into the integrated screening device to assay xylanase activity as described above. To generate the heat shock, the droplets were run through PTFE tubing (0.56 mm × 1.07 mm) immersed in a 90 °C water bath. The flow rate was set such that residence time within the tubing was 30 s. The droplets were then collected in a glass vial and reloaded into the integrated screening device to assay post-heat-shock levels of xylanase activity.

#### Xylanase activity screening of mutant library in microtiter plate

The microtiter plate cultures were centrifuged, and the supernatants were transferred to new microtiter plates. Xylanase activity was assayed using the EnzChek Ultra Xylanase Assay Kit (Thermo Fisher Scientific, Villebon-sur-Yvette, France). Briefly, 5 μl of supernatant was diluted in 45 μl of buffer. 25 μl of substrate (50 μg/ml) was added, and the resulting fluorescence was monitored (Biotek Mx microtiter plate reader, $\lambda _{em}$ = 360 nm, $\lambda _{em}$ = 460 nm) under constant agitation for 30 min. Vmax was calculated using a five-point window. The supernantants were then exposed to a heat shock event (90 °C, 30 s) and placed on ice. Xylanase activity was assayed as described above.

## Additional files

**Additional file 1: Figure S1.** Description of the dropmaker device. Droplets were produced by flow-focusing of the aqueous stream (inlet a) with two streams of fluorinated oilcontaining surfactant (inlet b). The device was used to produce 20 pl droplets that were collected off-chip (outlet c). The width of the microfluidic channels was 20 μm.

**Additional file 2: Movie 1.** Encapsulation of *Y. lipolytica* cells in 20 pl droplets. Movie of the dropmaker device during droplets formation. A *Y. lipolytica* cell suspension was encapsulated in 20 pl droplets. Droplets were produced at 3000 Hz.

**Additional file 3: Movie 2.** Encapsulation of *Y. lipolytica* cells in 20 pl droplets. Movie of the dropmaker device during droplet collection. Single *Y. lipolytica* cells can be seen in the 20 pl droplets.

**Additional file 4: Figure S2.** Description of the integrated screening device. Droplets were loaded (inlet b) and spaced by two streams of fluorinated oil containing surfactant (inlet a). The relevant enzymatic substrate (inlet c) was injected into each droplet by applying an AC field (20kHz, 200 V_*pp*_). The droplet contents were then mixed and the oil was extracted to increase the packing degree of the emulsion (inlet d, panel 2). Droplets were then incubated along the delay line and again spaced with two streams of fluorinated oil (inlets e) before entering the sorting module. The fluorescence of each droplet was measured. By default, the droplets flowed in the channel with the lower hydrodynamic resistance (outlet f). Droplets could be sorted based fluorescence intensity by applying AC field pulses (30 kHz; 1200 V_*pp*_; 0.3-0.6 ms) and collected (outlet g).

**Additional file 5: Movie 3.** Loading of the droplets into the microfluidic screening device. Movie of the microfluidic screening device at the loading part. Droplets were loaded and spaced with two oil streams. Droplets containing *Y. lipolytica* cells that were cultivated overnight can be seen.

**Additional file 6: Movie 4.** Picoinjection of the fluorogenic substrate. Movie of the microfluidic screening device at the picoinjection part. The relevant fluorogenic substrate was injected in each droplet by applying an AC field (20kHz, 200 V_*pp*_).

**Additional file 7: Movie 5.** On-chip droplets incubation. Movie of the microfluidic screening device at the incubation part. The droplets were incubated on-chip along a delay line, allowing the enzymatic reaction to turnover.

**Additional file 8: Figure S3.** Poisson's law distribution during encapsulation process. The number of yeast cells per droplets (k) after the encapsulation process is following a Poisson distribution and depends on the initial average number of cells per droplets (λ) of the encapsulated cell suspension.

**Additional file 9: Figure S4.** Microfluidic library analysis. The two libraries A and B and the parental strain JMY4510 (*xlnc*) were analyzed using the microfluidic screening device before (a) and after (b) heat shock (90°C, 30 sec). On the left, 1D histograms show the number of droplets observed as a function of the xylanase activity (blue fluorescence) for the ternary emulsion containing, from top to bottom, JMY4510 (*xlnc*), library A and library B. The mean signal for the wild-type is indicated with a green line, and values >σ and >3σ are to the right of the red lines. Strains were considered to be positives when displaying a xylanase activity higher than (mean wild-type activity + 3σ). The tables on the right provide the statistics for each population, both at the droplet and the cell level.

**Additional file 10: Table S1.** Thermostable variants of endo-*β*-1,4-xylanase C. Xylanase activity (RFU average) before and after heat treatment (90 °C) and residual activity of the 6 thermoresistant clones.

**Additional file 11: Figure S5.** Sequence alignment. Clustal multiple sequence alignment of the endo-β-1,4-xylanase C amino acid sequence and the translated sequencesof the mutated XYNC in the six clones identified as thermoresistant. Mutations are underlined in yellow.

**Additional file 12: Table S2.** *Y. lipolytica* and *E. coli* strains used in this study.

**Additional file 13: Table S3.** List of primers used to check cassette integration at the specific Zeta locus.

**Additional file 14: Figure S6.** Construction of *xlnc *libraries using error-prone PCR. The construction of xlnc mutant libraries was obtained by PCR amplification in two steps. First step, the PCR fragment composed of Zeta1, URA3 marker and pTEF promoter was amplified using primers Zeta1for and epPCRrev using the JME2603 plasmid as a template (top figure) under normal PCR condition (PCR1). Then, the PCR fragment composed of *xlnc *and Zeta2 was amplified using primers epPCR-for and Zeta2rev and the JME2603 plasmid as a template (top figure) under epPCR mutagenic conditions (PCR2). Second step, a fusion PCR was performed under standard PCR conditions using an equimolar proportion of both fragments as templates and using primers Zeta1for and Zeta2rev (PCR3).
