# Supplementary figures and images for: Droplet-based microfluidic high-throughput screening of heterologous enzymes secreted by the yeast Yarrowia lipolytica
Source: Microb Cell Fact. 2017 Jan 31;16:18. doi: 10.1186/s12934-017-0629-5 (PMC5282883; doi:10.1186/s12934-017-0629-5)

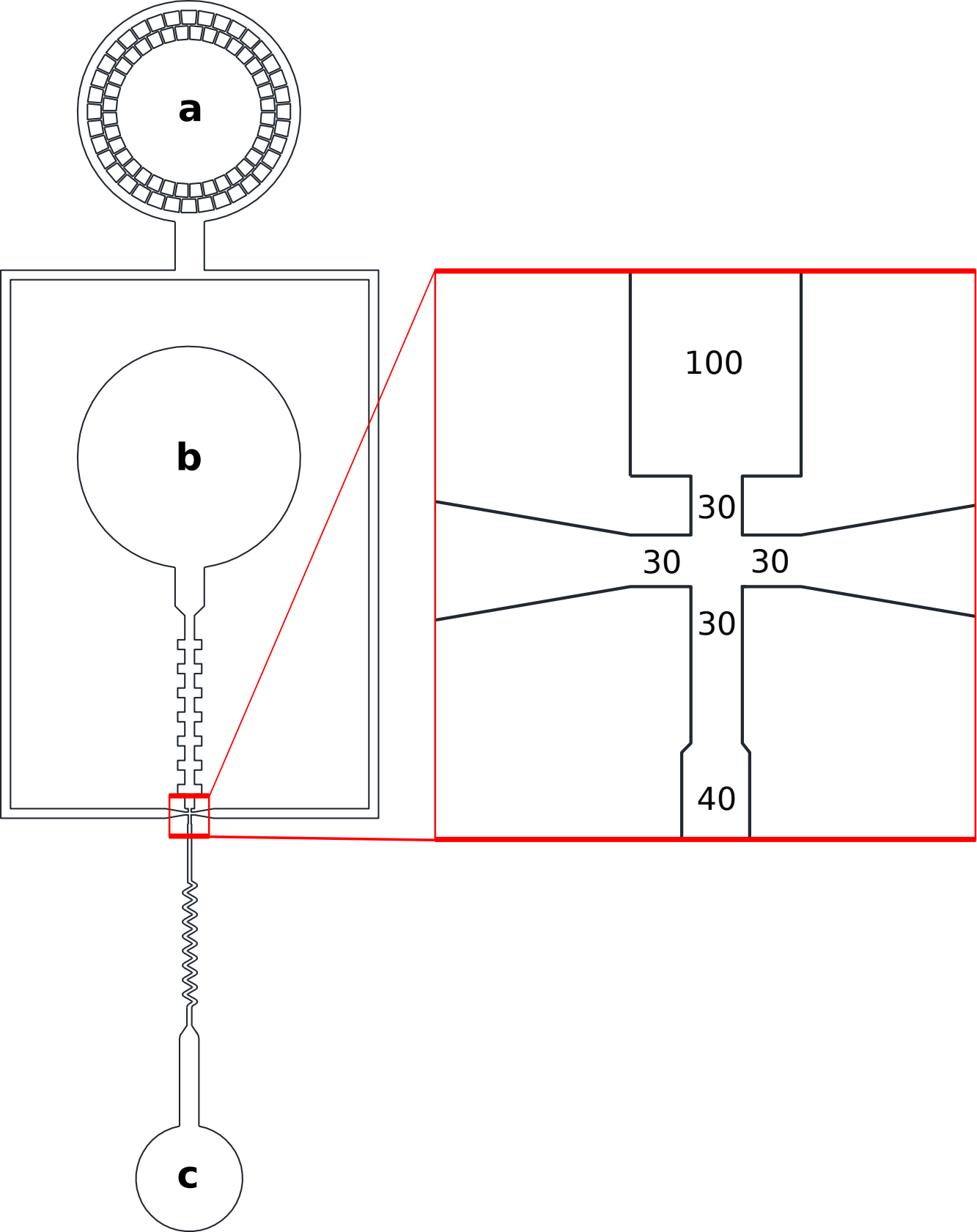

Supplement: Supplementary file 1 — Additional file 1: Figure S1. Description of the dropmaker device. Droplets were produced by flow-focusing of the aqueous stream (inlet a) with two streams of fluorinated oilcontaining surfactant (inlet b). The device was used to produce 20 pl droplets that were collected off-chip (outlet c). The width of the microfluidic channels was 20 μm. [file 12934_2017_629_MOESM1_ESM.pdf]

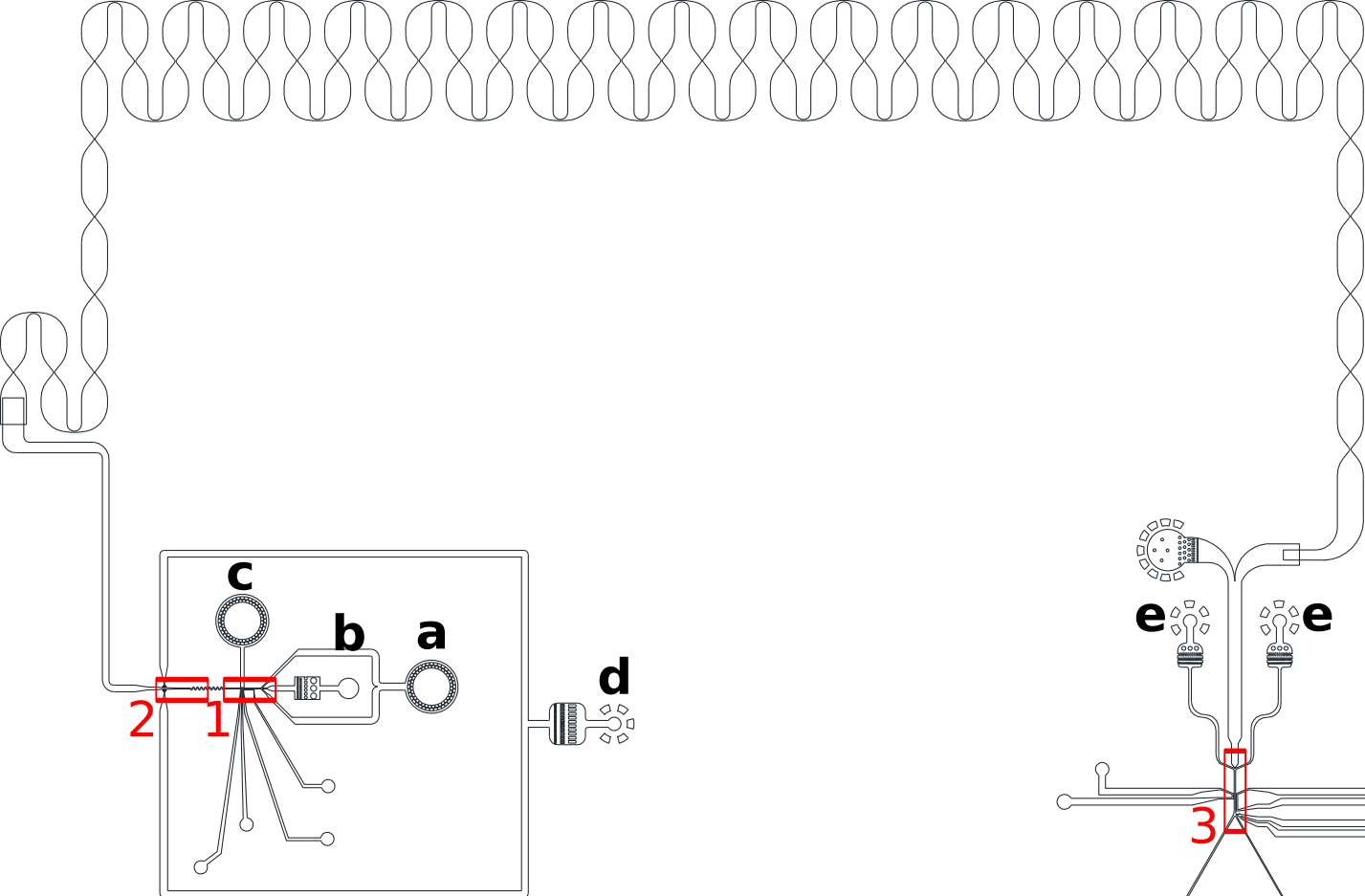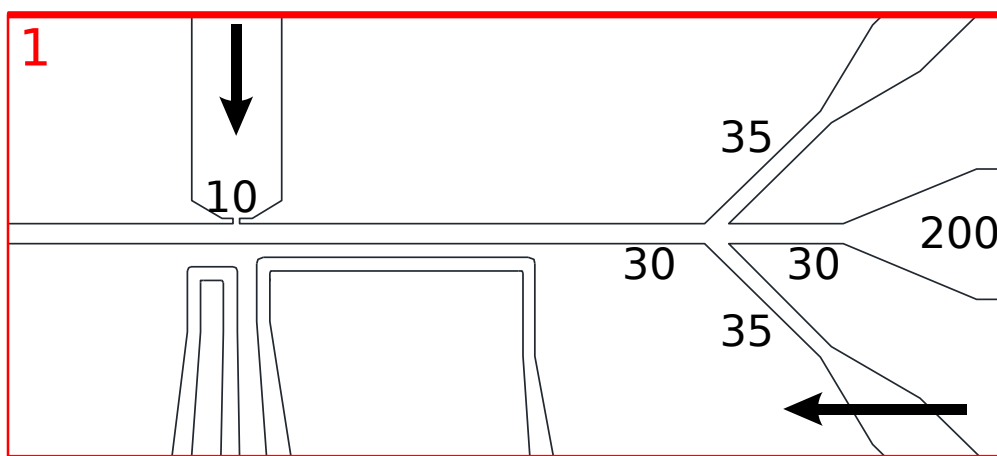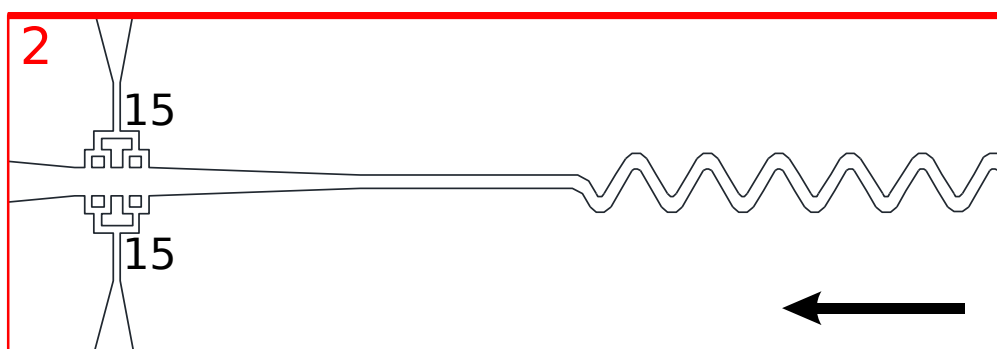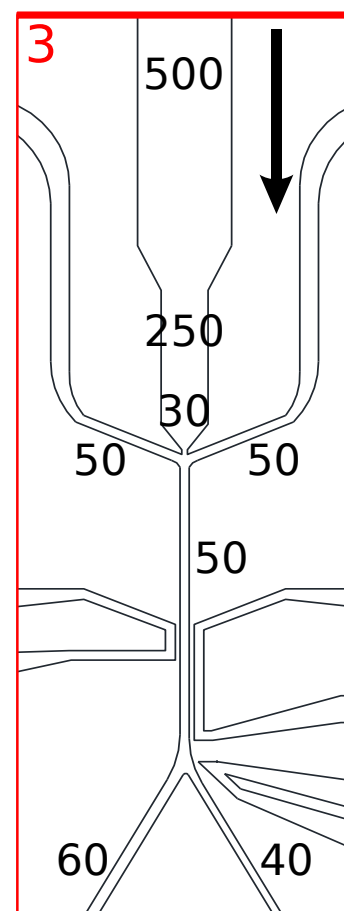

Supplement: Supplementary file 4 — Additional file 4: Figure S2. Description of the integrated screening device. Droplets were loaded (inlet b) and spaced by two streams of fluorinated oil containing surfactant (inlet a). The relevant enzymatic substrate (inlet c) was injected into each droplet by applying an AC field (20kHz, 200 Vpp). The droplet contents were then mixed and the oil was extracted to increase the packing degree of the emulsion (inlet d, panel 2). Droplets were then incubated along the delay line and again spaced with two streams of fluorinated oil (inlets e) before entering the sorting module. The fluorescence of each droplet was measured. By default, the droplets flowed in the channel with the lower hydrodynamic resistance (outlet f). Droplets could be sorted based fluorescence intensity by applying AC field pulses (30 kHz; 1200 Vpp; 0.3-0.6 ms) and collected (outlet g). [file 12934_2017_629_MOESM4_ESM.pdf]

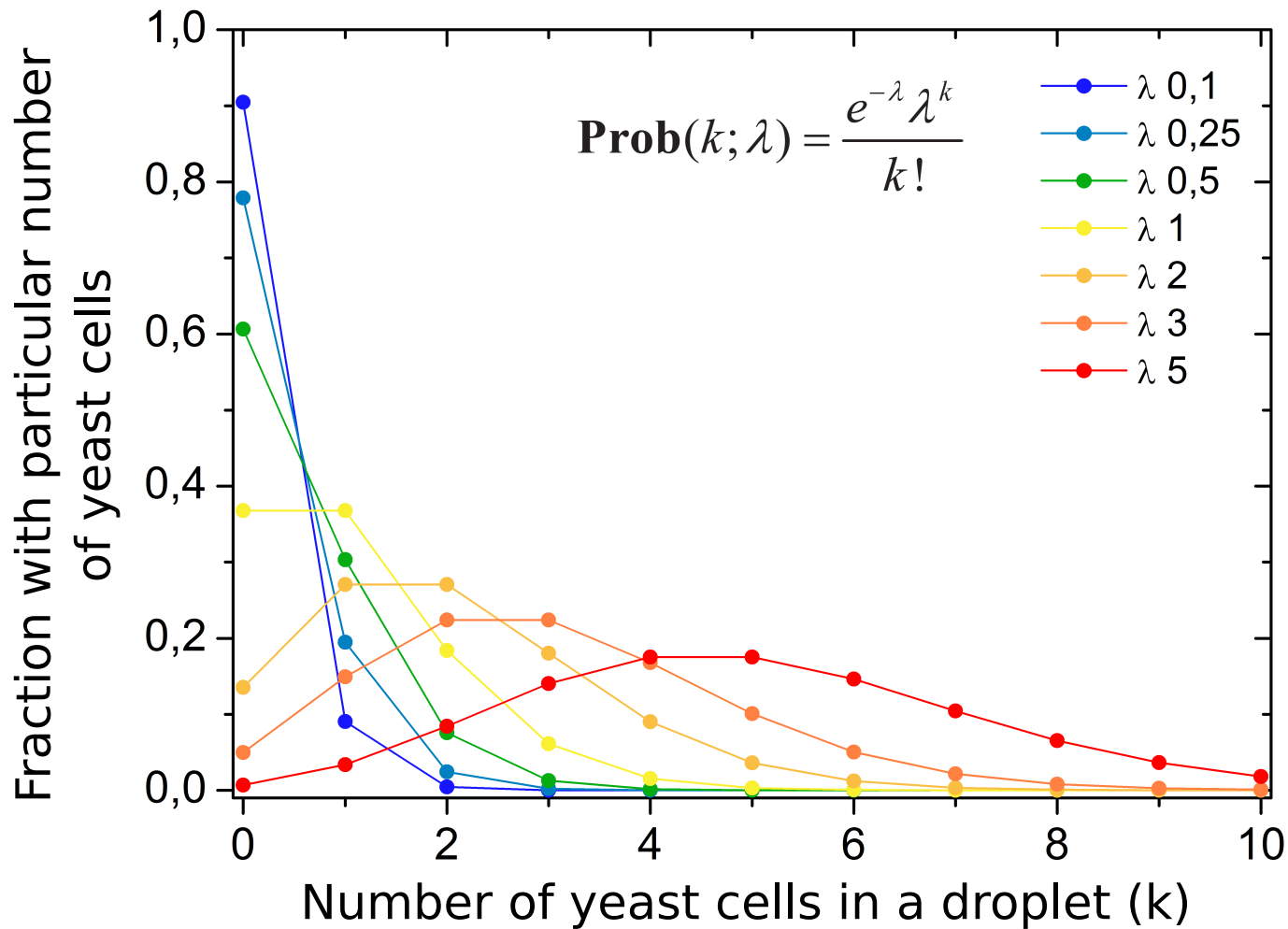

Supplement: Supplementary file 8 — Additional file 8: Figure S3. Poisson's law distribution during encapsulation process. The number of yeast cells per droplets (k) after the encapsulation process is following a Poisson distribution and depends on the initial average number of cells per droplets (λ) of the encapsulated cell suspension. [file 12934_2017_629_MOESM8_ESM.pdf]

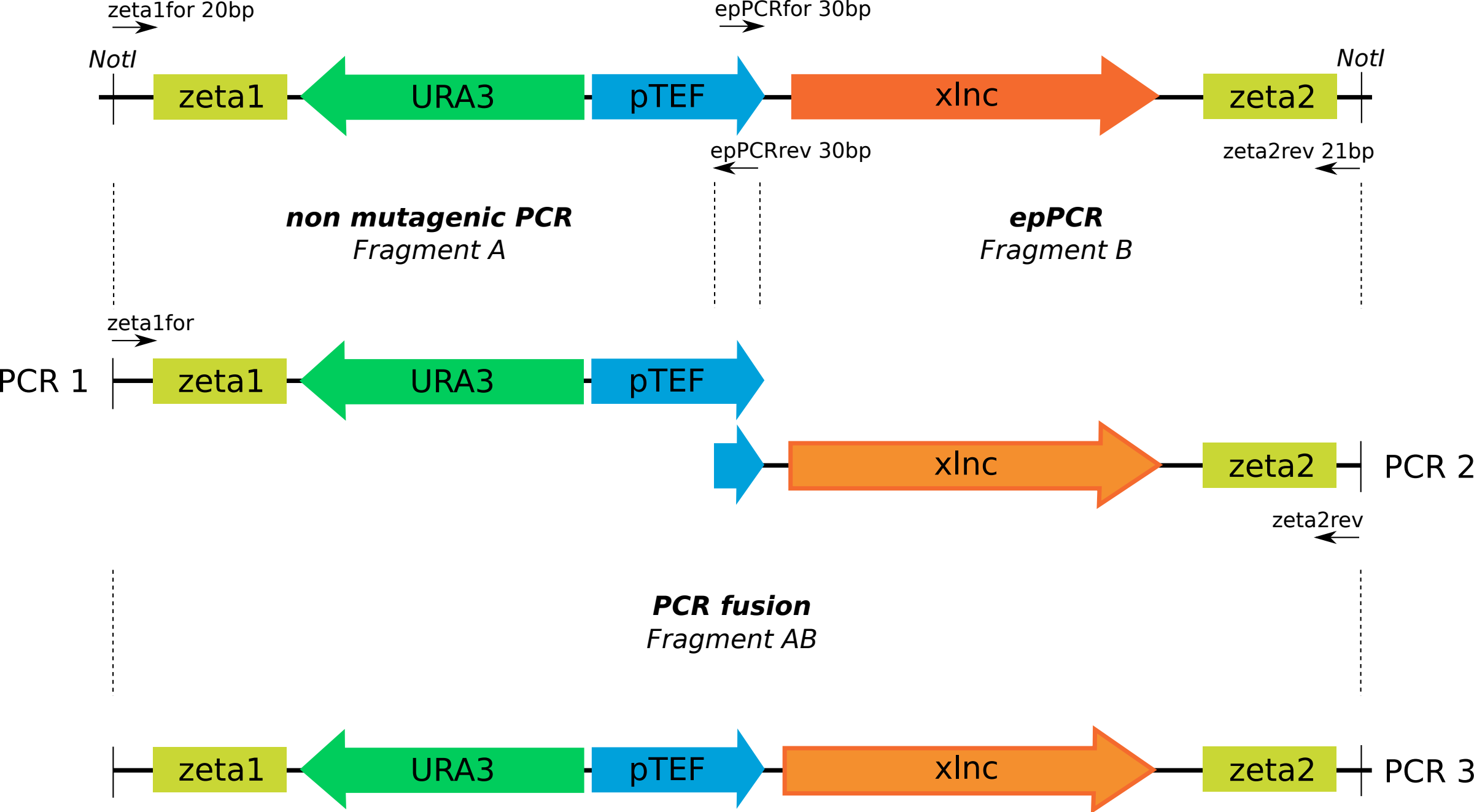

Supplement: Supplementary file 14 — Additional file 14: Figure S6. Construction of xlnc libraries using error-prone PCR. The construction of xlnc mutant libraries was obtained by PCR amplification in two steps. First step, the PCR fragment composed of Zeta1, URA3 marker and pTEF promoter was amplified using primers Zeta1for and epPCRrev using the JME2603 plasmid as a template (top figure) under normal PCR condition (PCR1). Then, the PCR fragment composed of xlnc and Zeta2 was amplified using primers epPCR-for and Zeta2rev and the JME2603 plasmid as a template (top figure) under epPCR mutagenic conditions (PCR2). Second step, a fusion PCR was performed under standard PCR conditions using an equimolar proportion of both fragments as templates and using primers Zeta1for and Zeta2rev (PCR3). [file 12934_2017_629_MOESM14_ESM.pdf]
