# Supplementary material for: Droplet-based microfluidic high-throughput screening of heterologous enzymes secreted by the yeast Yarrowia lipolytica
Source: Microb Cell Fact. 2017 Jan 31;16:18. doi: 10.1186/s12934-017-0629-5 (PMC5282883; doi:10.1186/s12934-017-0629-5)
Supplement: Supplementary file 9 — Additional file 9: Figure S4. Microfluidic library analysis. The two libraries A and B and the parental strain JMY4510 (xlnc) were analyzed using the microfluidic screening device before (a) and after (b) heat shock (90°C, 30 sec). On the left, 1D histograms show the number of droplets observed as a function of the xylanase activity (blue fluorescence) for the ternary emulsion containing, from top to bottom, JMY4510 (xlnc), library A and library B. The mean signal for the wild-type is indicated with a green line, and values >σ and >3σ are to the right of the red lines. Strains were considered to be positives when displaying a xylanase activity higher than (mean wild-type activity + 3σ). The tables on the right provide the statistics for each population, both at the droplet and the cell level. [file 12934_2017_629_MOESM9_ESM.pdf]

a

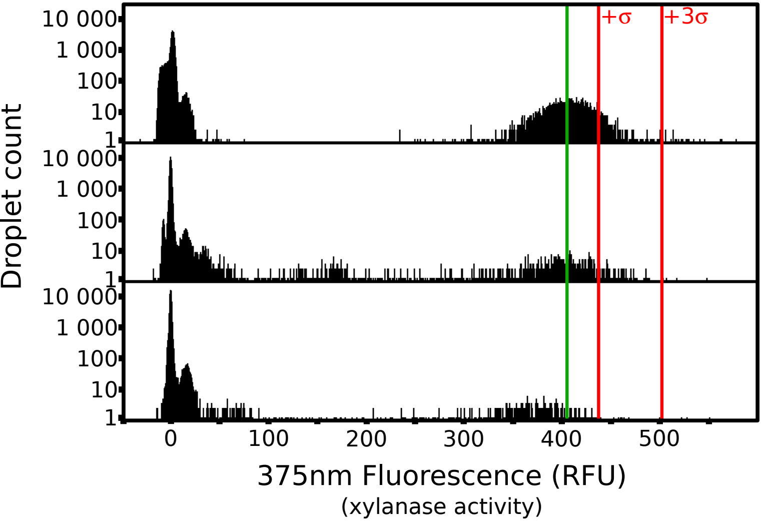

	**4510**	Library A	Library B
**Emulsion analysis**			
Number of analyzed droplets	61 500	66 500	100 000
Droplets occupancy	5.1%	7.0%	6.7%
Number of positive droplets	*3 089 (5.0%)*	*1 258 (1.89%)*	*590 (0.59%)*
>σ	*295 (0.48%)*	*113 (0.17%)*	*13 (0.013%)*
>3σ	*23 (0.037%)*	*4 (0.006%)*	*4 (0.004%)*
**Libraries analysis**			
Number of analyzed clones	3 136	4 660	6 625
Diversity	-	800	600
Number of positive clones	*3 089 (98.5%)*	*216 (27%)*	*54 (9%)*
>σ	*295 (9.4%)*	*19 (2.4%)*	*1 (0.17%)*
>3σ	*23 (0.73%)*	*<1 (0.09%)*	*<1 (0.06%)*

b

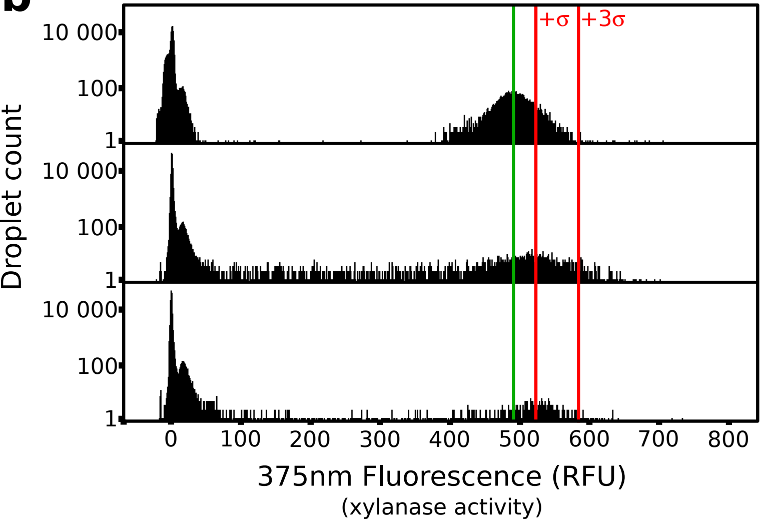

	**4510**	Library A	Library B
**Emulsion analysis**			
Number of analyzed droplets	165 000	206 000	230 000
Droplets occupancy	5.1%	7.0%	6.7%
Number of positive droplets	8 213 (5.0%)	2 884 (1.4%)	966 (0.42%)
>σ	793 (0.48%)	784 (0.38%)	253 (0.11%)
>3σ	26 (0.016%)	160 (0.078%)	36 (0.015%)
**Libraries analysis**			
Number of analyzed clones	8 415	14 420	15 410
Diversity	-	800	600
Number of positive clones	*8 213 (97.5%)*	*160 (20%)*	*37 (6.1%)*
>σ	*793 (9.4%)*	*43 (5.4%)*	*9 (1.5%)*
>3σ	*26 (0.003%)*	*8 (1%)*	*1 (0.17%)*
