# Supplementary material for: Droplet-based microfluidic high-throughput screening of heterologous enzymes secreted by the yeast Yarrowia lipolytica
Source: Microb Cell Fact. 2017 Jan 31;16:18. doi: 10.1186/s12934-017-0629-5 (PMC5282883; doi:10.1186/s12934-017-0629-5)
Supplement: Supplementary file 11 — Additional file 11: Figure S5. Sequence alignment. Clustal multiple sequence alignment of the endo-β-1,4-xylanase C amino acid sequence and the translated sequencesof the mutated XYNC in the six clones identified as thermoresistant. Mutations are underlined in yellow. [file 12934_2017_629_MOESM11_ESM.pdf]

```
XYNC      MVQIKVAALAMLFASQVLSEPIEPRQASVSIDTKFKAHGKKYLGNIGDQYTLTKNSKTPA
Clone1    MEQIKVAALAMLFASQVLSEPIEPRQASVSIDTKFKAHGKKYLGNIGDQYTLTKNSKTPA
Clone2    MVQIKVAALAMLFASQVLSEPIEPRRASVSIDTKFKAHGKKYLGNIGDQYTLTKNSKTPA
Clone3    MVQIKEAALAMLFASQVLSEPIEPRQASVSIDTKFKARGKKYLGNIGDQYTLTKNSKTPA
Clone4    MVQIKVAALAMLFASQVLSEPIEPRQASVSIDTKFKAHGKKYLGNIGDQYTLTKNSKTPA
Clone5    MVQIKVAALATLFASQVLSEPIEPRQASVSIDTKFKAHGKKYLGNIGDQYTLAKNSKTPA
Clone6    MVQIKVAALAMLFASQVSSEPIEPRQASVSIDTKFKAHGKKYLGNIGDQYTLTKNSKTPT
          * *** **** ****** ********:**********:**************:******:

XYNC      IIKADFGALTPENSMKWDATEPSRGQFSFSGSDYLVNFAQSNNKLIRGHTLVWHSQLPSW
Clone1    IIKADFGALTPENSMKWDATEPSRGQFSFSGSDYLVNFAQSNNKLIRGHTLVWHSQLPSW
Clone2    IIKADFGALTPENSMKWDATEPSRGQFSFSGSDYLVNXAQSNNKLIRGHTLVWHSQLPSW
Clone3    IIKADFGALTPENSMKWDATEPSRGQFSFSGSDYLVNFAQSNNKLIRGHTLVWHSQLPSW
Clone4    IIKADFGALTPENSMKWDATEPSRGQFSFSGSDYLVNFAQSNNKLIRGHTLVWHSQLPSW
Clone5    IIRADFGALTPENSMKWDATEXSRGQFSFSGSDYLVNFAQSNNKLIRGHTLVWHSQLPSW
Clone6    IIKADFGALTPENSMKWDATEPSRGQFSFSGSDYLVNFAQSNNKLIRGHTLVWHSQLPSW
          **:****************** *************** **********************

XYNC      VQSITDKNTLIEVMKNHITTVMQHYKGKIYAWDVVNEIFNEDGSLRDSVFYKVIGEDYVR
Clone1    VQSITDKNTLIEVMKNHITTVMQHYKGKIYAWDVVNEIFNEDGSLRDSVFYKVIGEDYVR
Clone2    VQSITDKNTLIEVMKNHITTVMQHYKGKIYAWDVVNEIFNEDGSLRDSVFYKVIGEDYVR
Clone3    VQSITDKNTLIEVMKNHITTVMQHYKGKIYAWDVVNEIFNEDGSLRDSVFYKVIGEDYVR
Clone4    VQSITDKNTLIEVMKNHITTVMQHYKGKIYAWDVVNEIFNEDGSLRDSVFYKVIGEDYVR
Clone5    IQSITDKNTLIEVMKNHITTVMQHYKGKIYAWDVVNEIFNEDGSLRDSVFYKVIGEDYVR
Clone6    VQSITDKNTLIEVMKNHITTVMQHYKGKIYAWDVVNEIFNEDGSLRDSVFYKVIGEDYVR
          :***********************************************************

XYNC      IAFETARAADPNAKLYINDYNLDSASYPKLTGMVSHVKKWIAAGIPIDGIGSQTHLSAGG
Clone1    IAFETARAADPNAKLYINDYNLDSASYPKLTGMVSHVKKWIAAGIPIDGIGSQTHLSAGG
Clone2    IAFETARAADPNAKLYINDYNLDSASYPKLTGMVSHVKKWIAAGIPIDGIGSQTHLSAGG
Clone3    IAFETARAADPNAKLYINDYNLDSASYPKLTGMVSHVKKWIAAGIPIDGIGSQTHLSAGG
Clone4    IAFETARAADPNAKLYINDYNLDSASYPKLTGMVSHVKKWIAAGIPIDGIGSQTHLSXGG
Clone5    IAFETARAADPNAKLYINDYNLDSASYPKLTGMVSHVKKWIAAGIPIDGIGSQTHLSAGG
Clone6    IAFETARAADPNAKLYINDYNLDSASYPKLTGMVSQVKKWIAAGIPIDGIGSQTHLSAGG
          ***********************************:********************* **

XYNC      GAGISGALNALAGAGTKEIAVTELDIAGASSTDYVEVVEACLNQPKCIGITVWGVADPDS
Clone1    GAGISGALNALAGAGTKEIAVTELDIAGASSTDYVEVVEACLNQPKCIGITVWGVADPDS
Clone2    GAGISGALNALAGAGTKEIAVTELDIAGASSTDYVEVVEACLNQPKCIGITVWGVADPDS
Clone3    GAGISGALNALAGAGTKEIAVTELDIAGASSTDYVEVVEACLNQPKCIGITVWGVADPDS
Clone4    GAGISGALNALAGAGTKEIAVTELDIAGASSTDYVEVVEACLNQPKCIGITVWGVADPDS
Clone5    GAGISGALNALAGAGTKEIAVTELDIAGASSTDYVDVVEACLNQPKCIGITVWGVADPDS
Clone6    GAGISGALNALAGAGTKEIAVTELDIAGASSTDYVEVVEACLNQPKCIGITVWGVADPDS
          ***********************************:************************

XYNC      WRSSSTPLLFDSNYNPKPAYTAIANAL*
Clone1    WRSSSTPLLFDSNYNPKPAYTAIANAL*
Clone2    WRSSSTPLLFDSNYNPKPAYTAIANAL*
Clone3    WRSSSTPLLFDSNYNPKPAYTAIANAL*
Clone4    WRSSSTPLLFDSNYNPKPAYAAIANAL*
Clone5    WRSSSTPLLFDSNYNPKPAYTAIANAL*
Clone6    WRSSSTPLLFDSNYNPKPAYTAIANAL*
          ********************:*******
```
